# Supplementary figures and images for: Longitudinal TprK profiling of in vivo and in vitro-propagated Treponema pallidum subsp. pallidum reveals accumulation of antigenic variants in absence of immune pressure
Source: PLoS Negl Trop Dis. 2021 Sep 7;15(9):e0009753. doi: 10.1371/journal.pntd.0009753 (PMC8480903; doi:10.1371/journal.pntd.0009753)

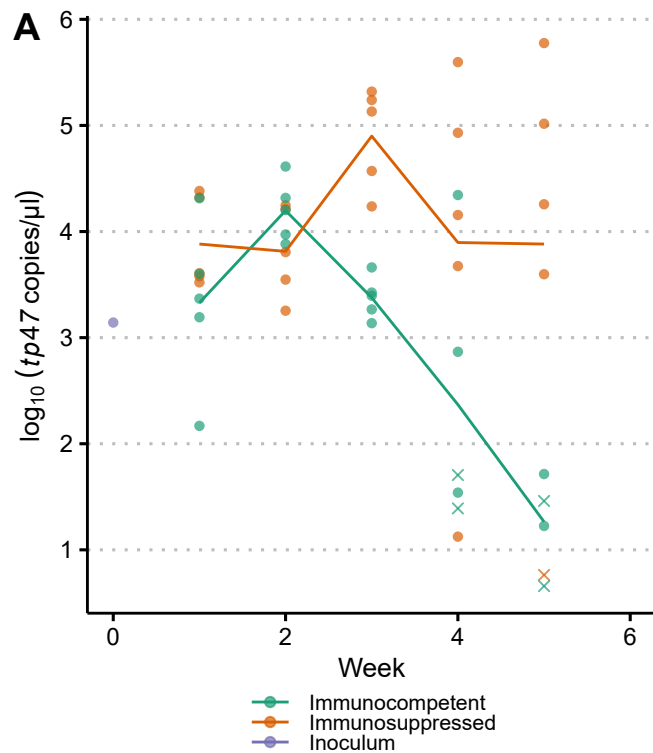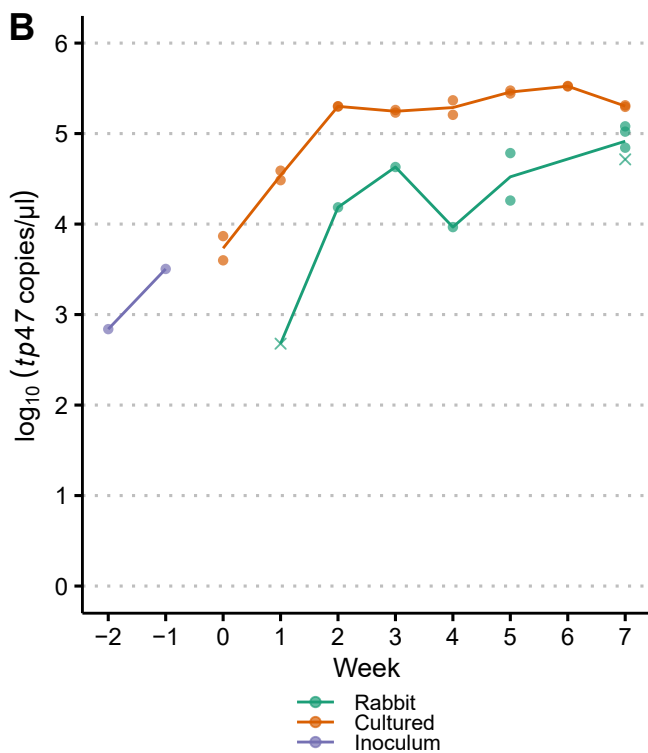

Supplement: S1 Fig — Each unique sample is represented by either a dot or a cross, where a cross indicates that we were unable to successfully recover sequence from the sample. Lines show the mean treponemal load, as quantified by the log of tp0574/μl, and are grouped and color-coded by passage type. (PDF) [file pntd.0009753.s004.pdf]

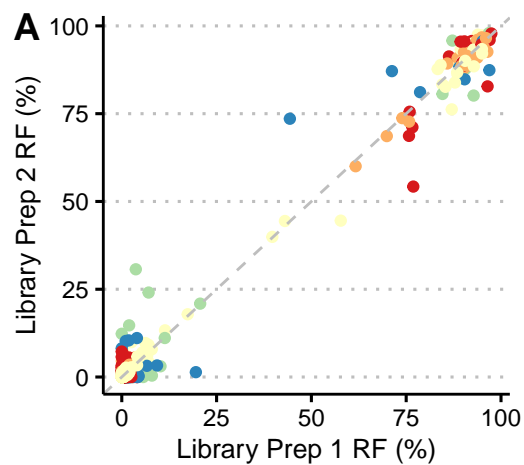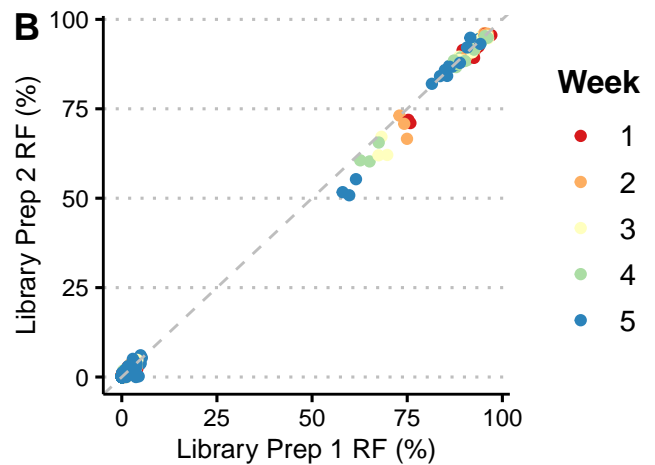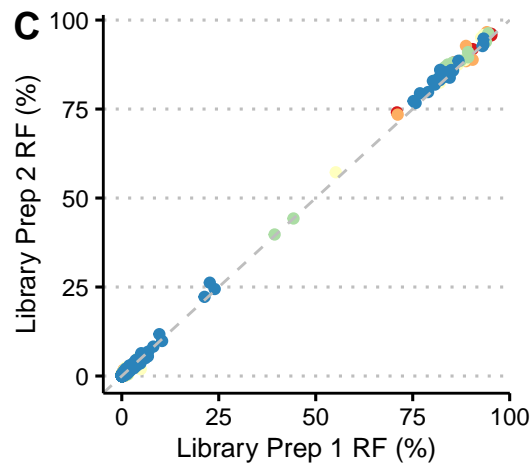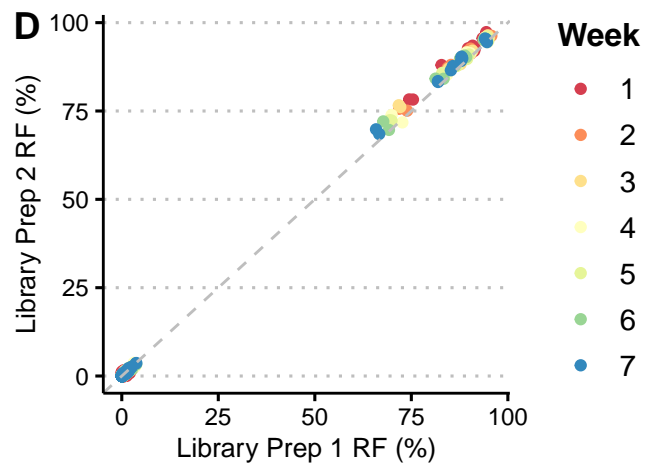

Supplement: S2 Fig — (PDF) [file pntd.0009753.s005.pdf]

# Culture A

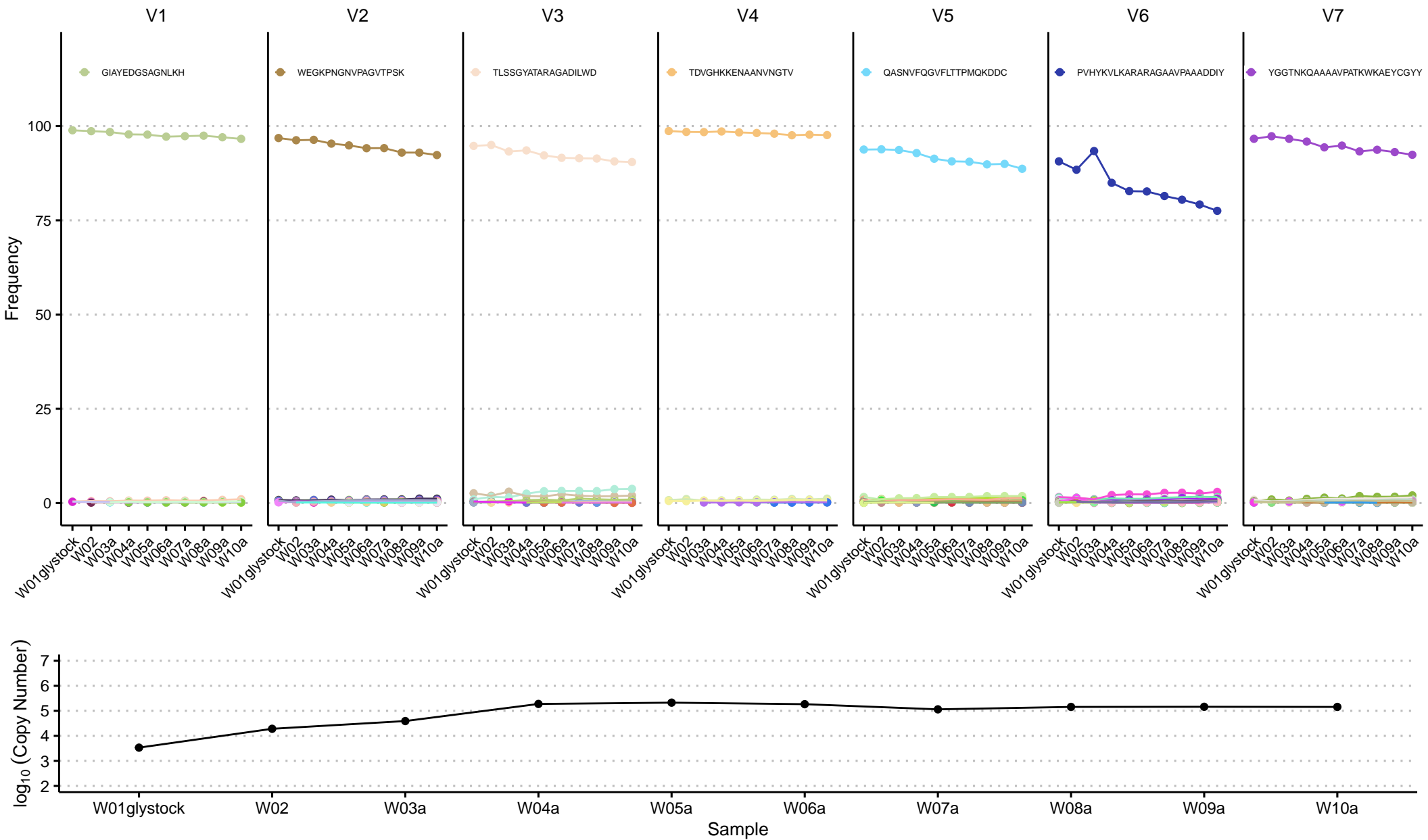

# Culture B

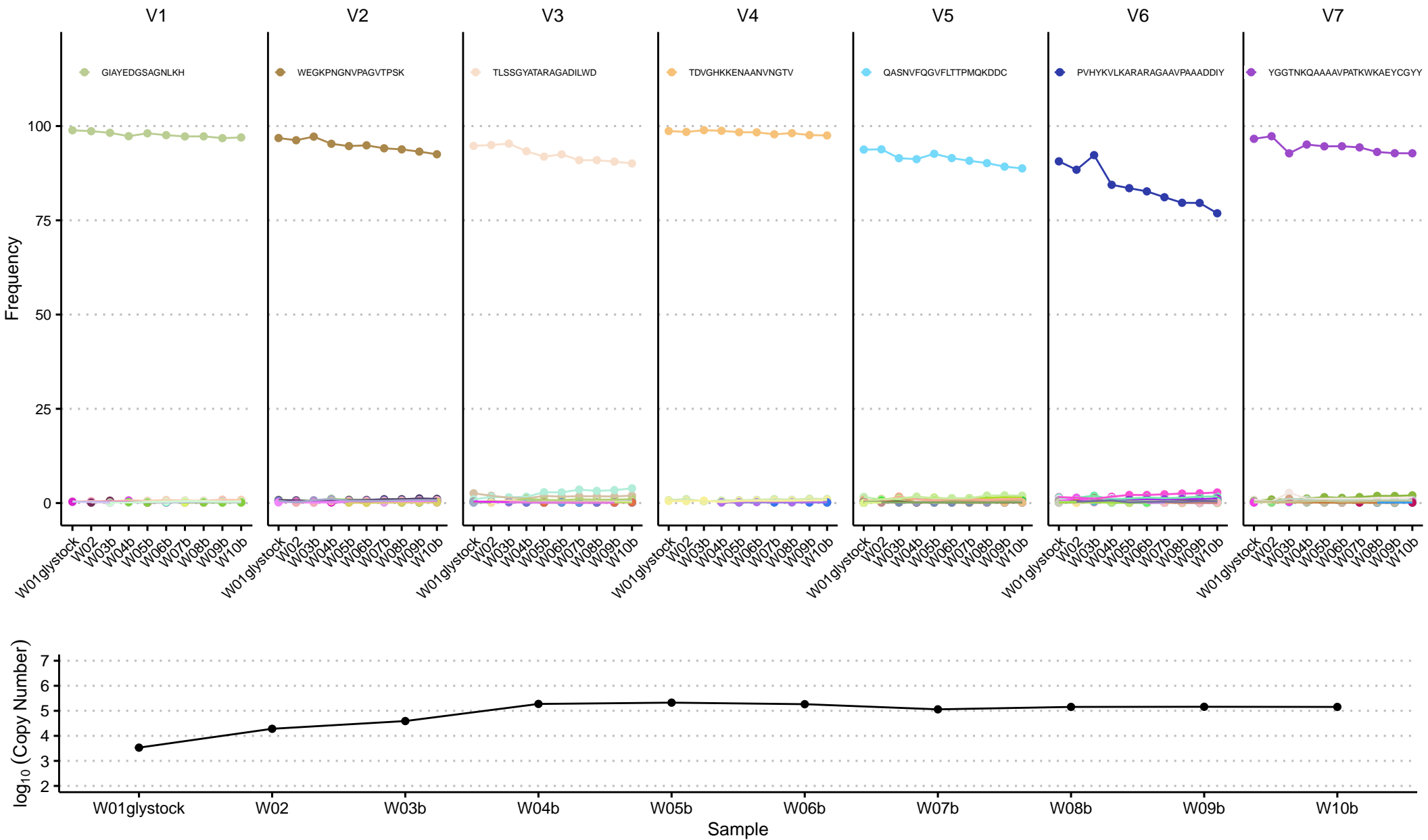

# Rabbit

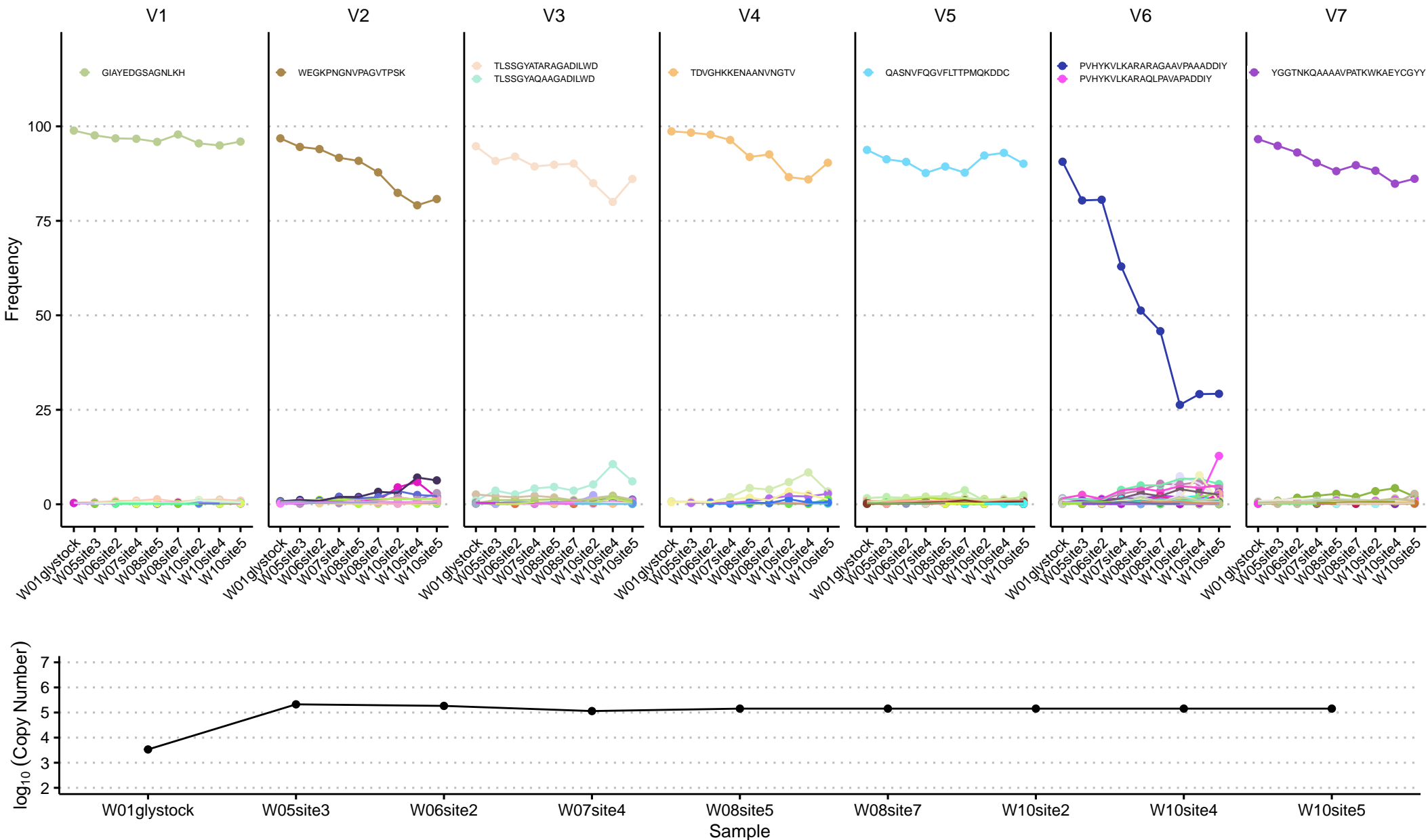

Supplement: S4 Fig — Only tprK alleles existing across multiple collection timepoints are present. Alleles that have >10% frequency at any time point are specifically labeled at the top of each variable region. Titles at top indicate immunosuppression status, and samples are grouped together for ease of comparison. (PDF) [file pntd.0009753.s007.pdf]

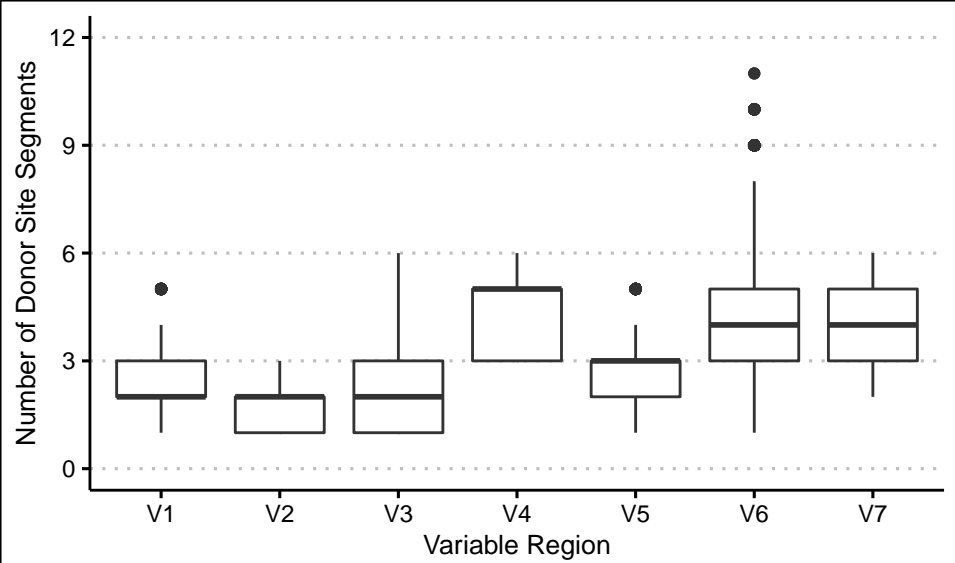

Supplement: S6 Fig — Internal 4-bp repeats are not included in the number of donor site segments. All combination possibilities with the highest % coverage were kept. Outliers are shown as dots outside of the boundaries of each boxplot. (PDF) [file pntd.0009753.s009.pdf]
