# Supplementary material for: Longitudinal TprK profiling of in vivo and in vitro-propagated Treponema pallidum subsp. pallidum reveals accumulation of antigenic variants in absence of immune pressure
Source: PLoS Negl Trop Dis. 2021 Sep 7;15(9):e0009753. doi: 10.1371/journal.pntd.0009753 (PMC8480903; doi:10.1371/journal.pntd.0009753)
Supplement: S3 Fig — Only tprK alleles existing across multiple collection timepoints are present. Alleles that have >10% frequency at any time point are specifically labeled at the top of each variable region. Titles at top indicate immunosuppression status, and samples are grouped together for ease of comparison. (PDF) [file pntd.0009753.s006.pdf]

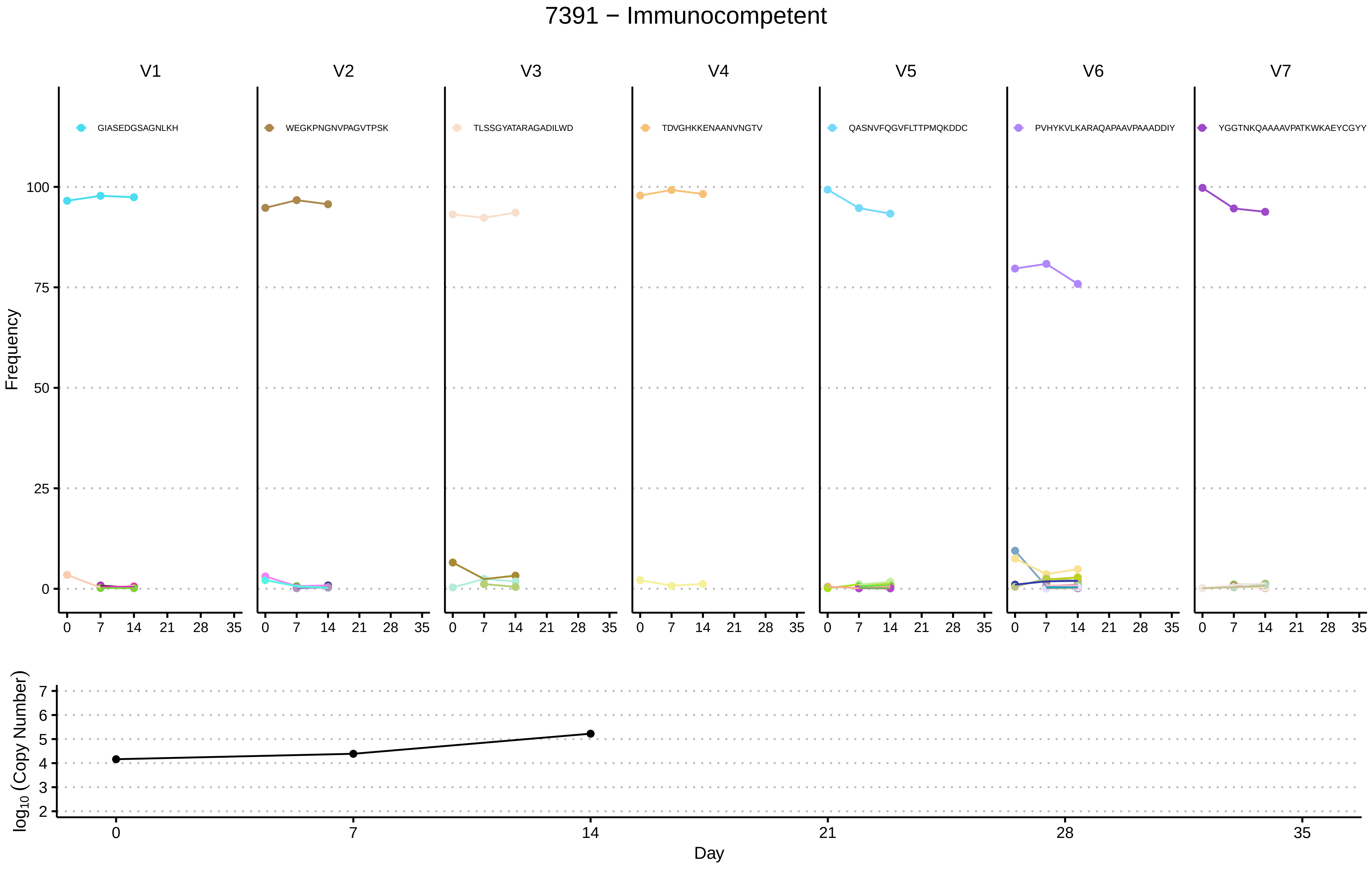

7391 − Immunocompetent
V1
V2
V3
V4
V5
V6
V7
GIASEDGSAGNLKH
WEGKPNGNVPAGVTPSK
TLSSGYATARAGADILWD
TDVGHKKENAANVNGTV
QASNVFQGVFLTTPMQKDDC
PVHYKVLKARAQAPAAVPAAADDIY
YGGTNKQAAAAVPATKWKAEYCGYY
Frequency
0
25
50
75
100
0
7
14
21
28
35
log10 (Copy Number)
2
3
4
5
6
7
Day

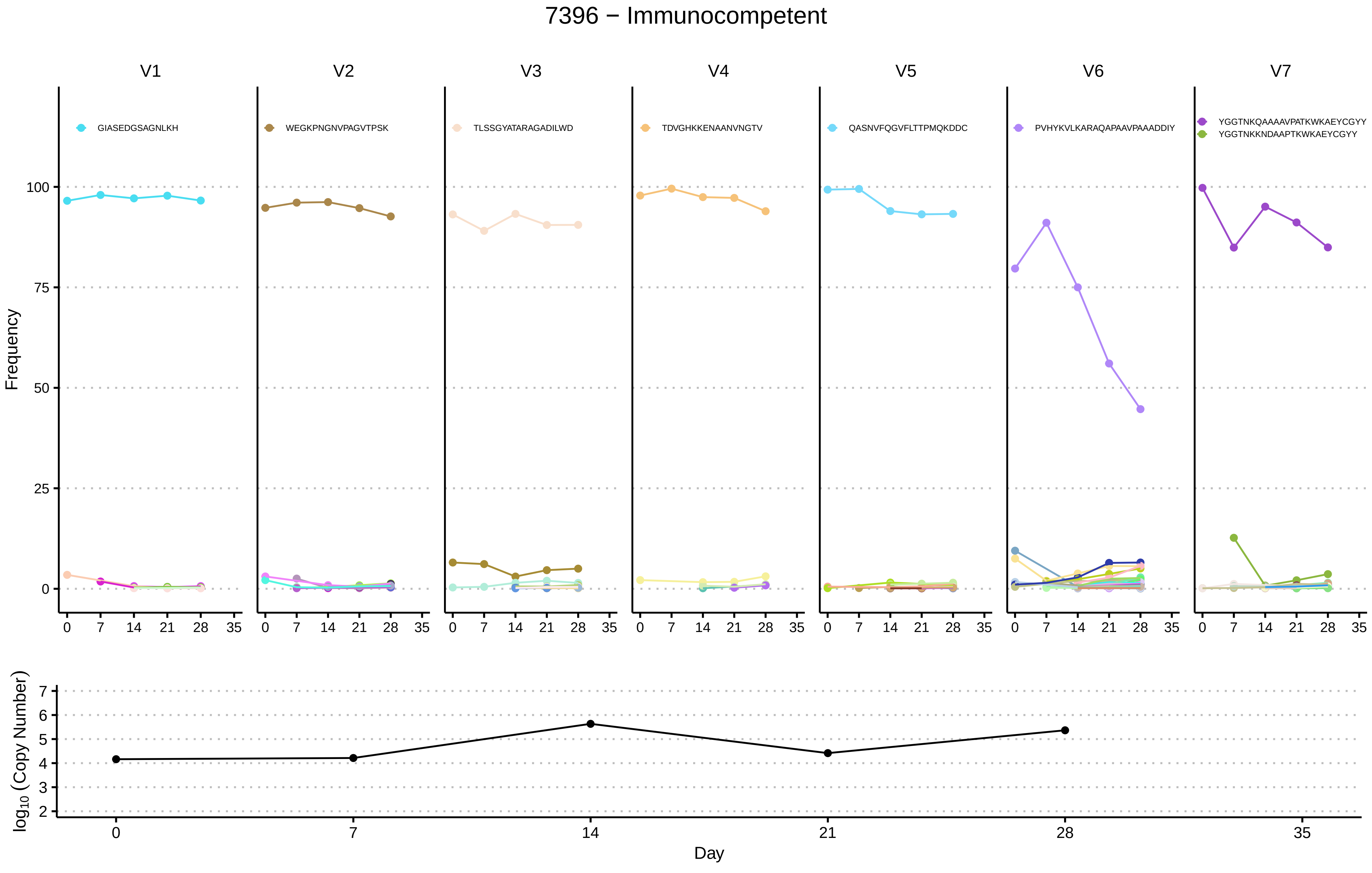

7396 − Immunocompetent
V1
V2
V3
V4
V5
V6
V7
GIASEDGSAGNLKH
WEGKPNGNVPAGVTPSK
TLSSGYATARAGADILWD
TDVGHKKENAANVNGTV
QASNVFQGVFLTTPMQKDDC
PVHYKVLKARAQAPAAVPAAADDIY
YGGTNKQAAAAVPATKWKAEYCGYY
YGGTNKKNDAAPTKWKAEYCGYY
Frequency
100
75
50
25
0
0 7 14 21 28 35
log10 (Copy Number)
7
6
5
4
3
2
0
7
14
21
28
35
Day

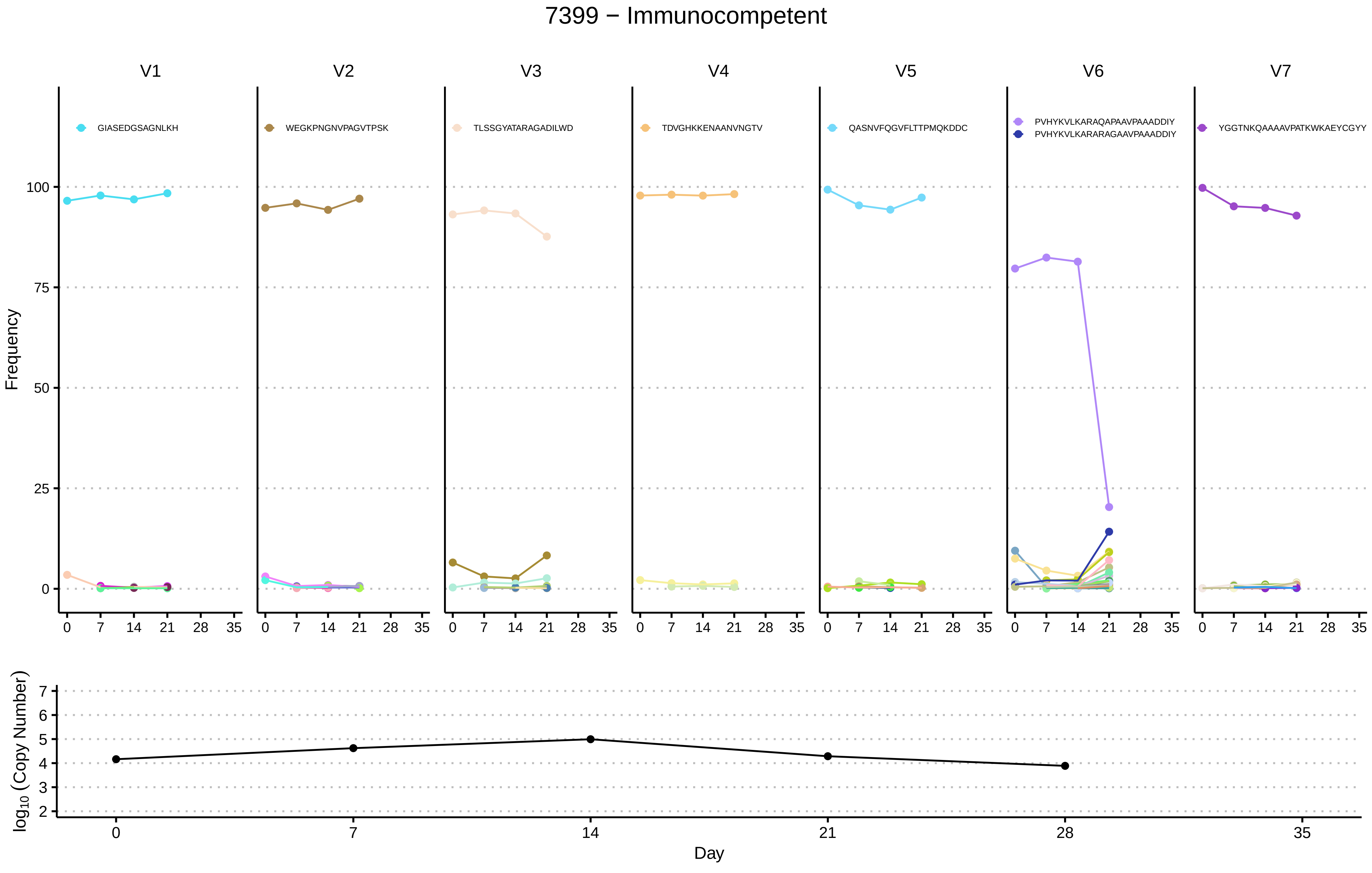

7399 − Immunocompetent
V1
V2
V3
V4
V5
V6
V7
GIASEDGSAGNLKH
WEGKPNGNVPAGVTPSK
TLSSGYATARAGADILWD
TDVGHKKENAANVNGTV
QASNVFQGVFLTTPMQKDDC
PVHYKVLKARAQAPAAVPAAADDIY
PVHYKVLKARARAGAAVPAAADDIY
YGGTNKQAAAAVPATKWKAEYCGYY
Frequency
100
75
50
25
0
0 7 14 21 28 35
log10 (Copy Number)
7
6
5
4
3
2
Day

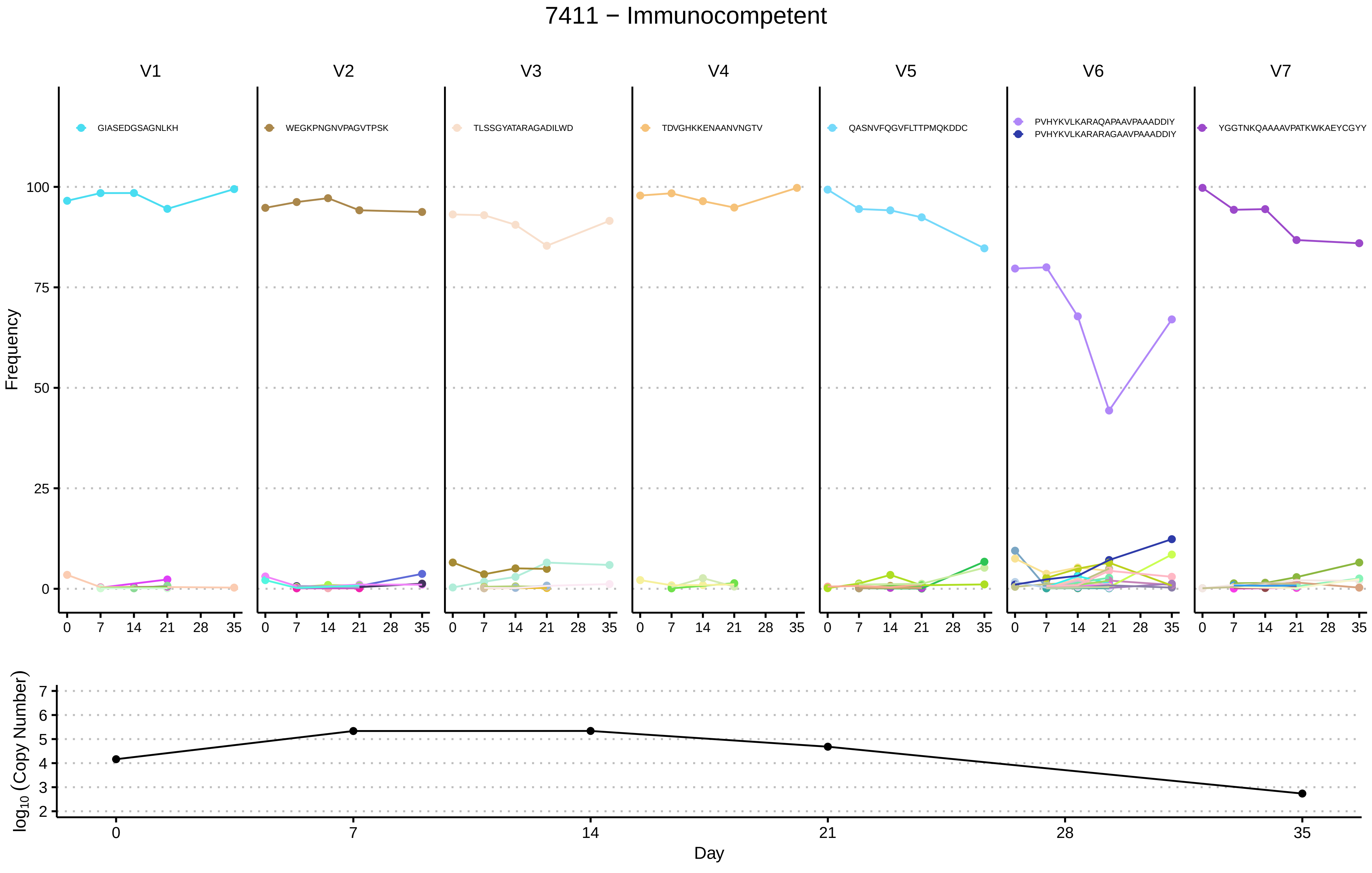
7411 − Immunocompetent
V1
V2
V3
V4
V5
V6
V7
GIASEDGSAGNLKH
WEGKPNGNVPAGVTPSK
TLSSGYATARAGADILWD
TDVGHKKENAANVNGTV
QASNVFQGVFLTTPMQKDDC
PVHYKVLKARAQAPAAVPAAADDIY
PVHYKVLKARARAGAAVPAAADDIY
YGGTNKQAAAAVPATKWKAEYCGYY
Frequency
100
75
50
25
0
0 7 14 21 28 35
log10 (Copy Number)
7
6
5
4
3
2
Day

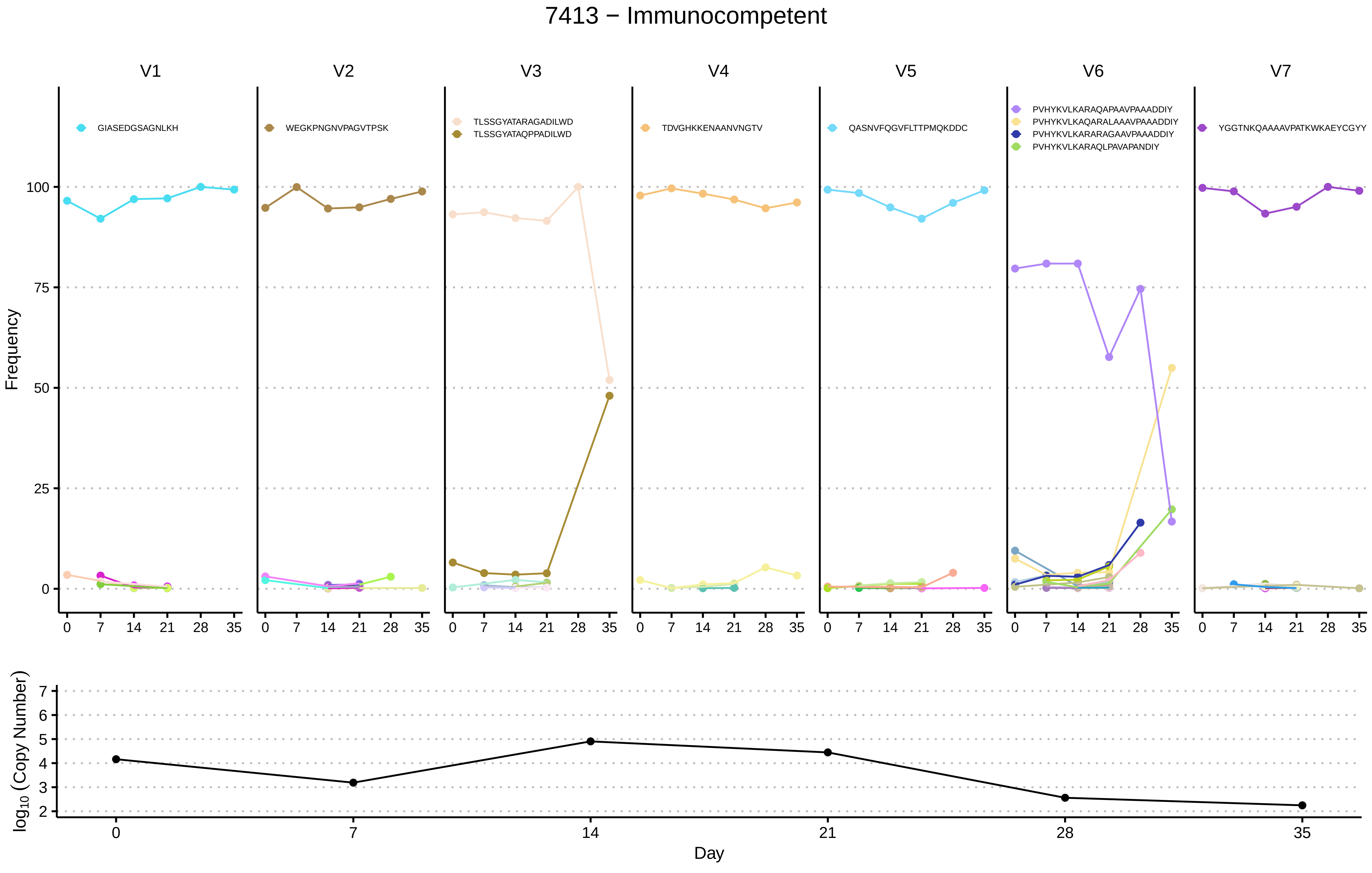
7413 − Immunocompetent
V1
V2
V3
V4
V5
V6
V7
GIASEDGSAGNLKH
WEGKPNGNVPAGVTPSK
TLSSGYATARAGADILWD
TLSSGYATAQPPADILWD
TDVGHKKENAANVNGTV
QASNVFQGVFLTTPMQKDDC
PVHYKVLKARAQAPAAVPAAADDIY
PVHYKVLKAQARALAAAVPAAADDIY
PVHYKVLKARARAGAAVPAAADDIY
PVHYKVLKARAQLPAVAPANDIY
YGGTNKQAAAAVPATKWKAEYCGYY
Frequency
0
25
50
75
100
0 7 14 21 28 35
$\log_{10}$ (Copy Number)
2
3
4
5
6
7
Day
0 7 14 21 28 35

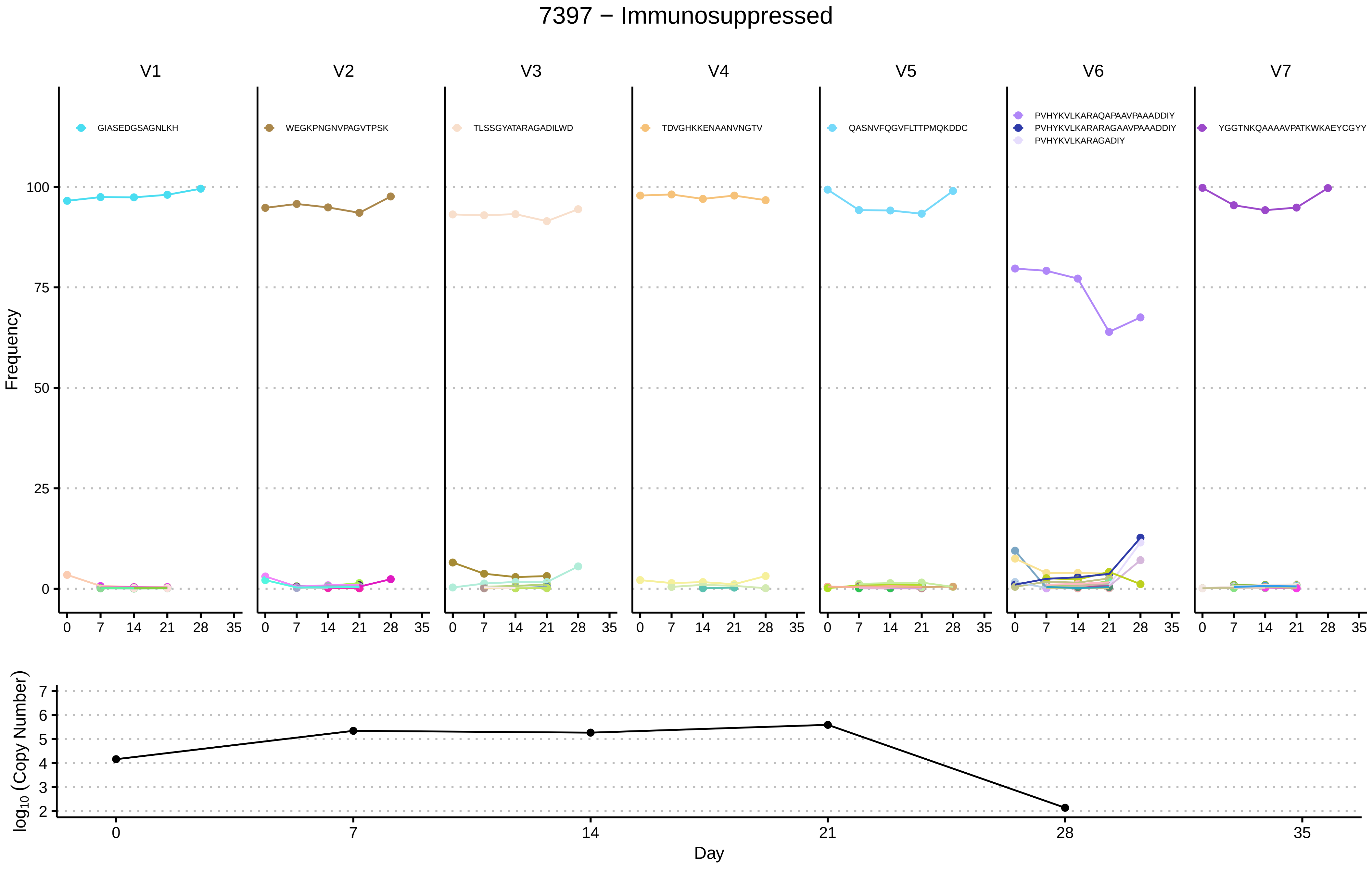
7397 − Immunosuppressed
V1
V2
V3
V4
V5
V6
V7
GIASEDGSAGNLKH
WEGKPNGNVPAGVTPSK
TLSSGYATARAGADILWD
TDVGHKKENAANVNGTV
QASNVFQGVFLTTPMQKDDC
PVHYKVLKARAQAPAAVPAAADDIY
PVHYKVLKARARAGAAVPAAADDIY
PVHYKVLKARAGADIY
YGGTNKQAAAAVPATKWKAEYCGYY
Frequency
100
75
50
25
0
0 7 14 21 28 35
log10 (Copy Number)
7
6
5
4
3
2
Day

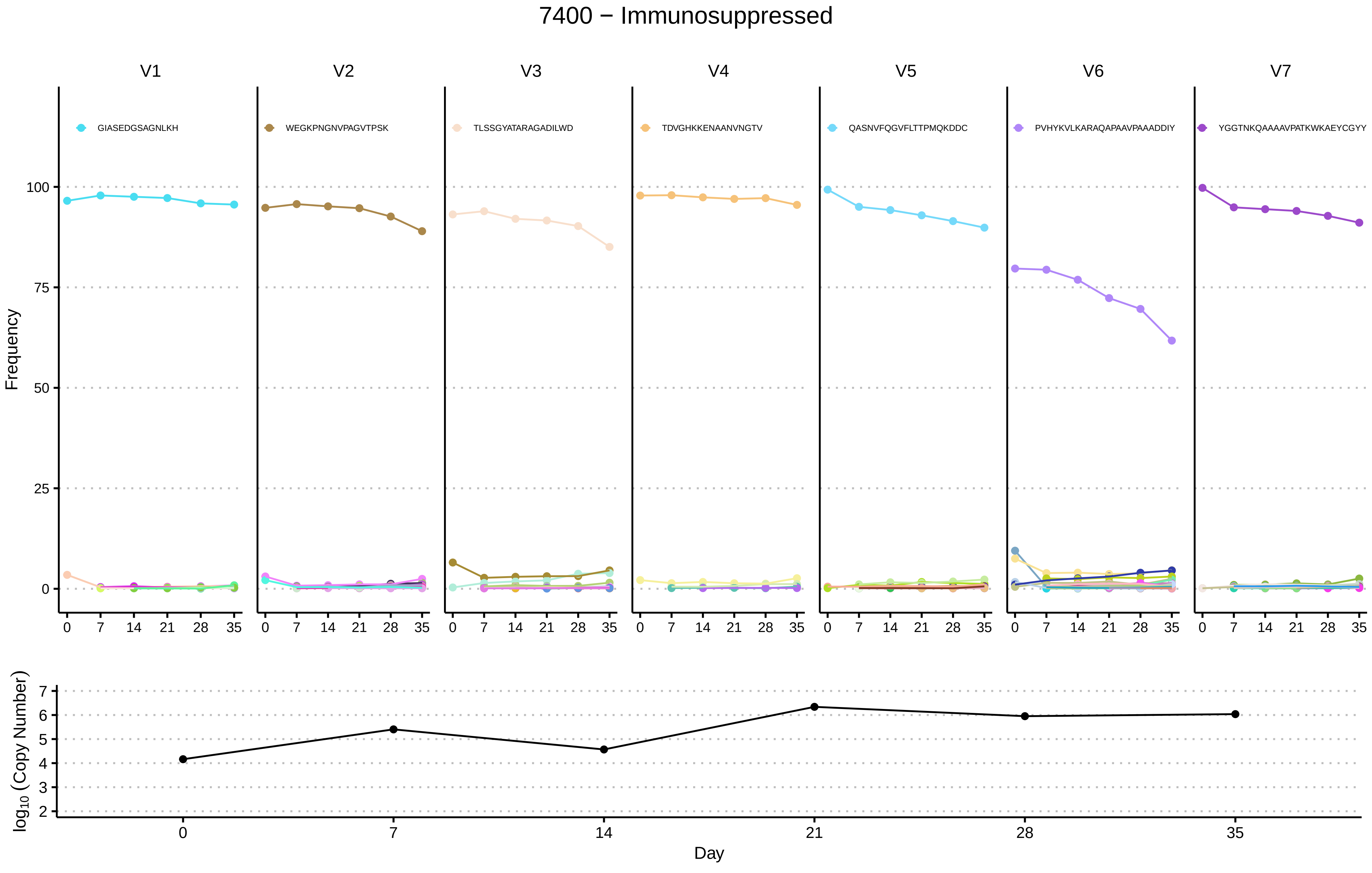

7400 − Immunosuppressed
V1
V2
V3
V4
V5
V6
V7
GIASEDGSAGNLKH
WEGKPNGNVPAGVTPSK
TLSSGYATARAGADILWD
TDVGHKKENAANVNGTV
QASNVFQGVFLTTPMQKDDC
PVHYKVLKARAQAPAAVPAAADDIY
YGGTNKQAAAAVPATKWKAEYCGYY
Frequency
100
75
50
25
0
0 7 14 21 28 35
log10 (Copy Number)
7
6
5
4
3
2
Day

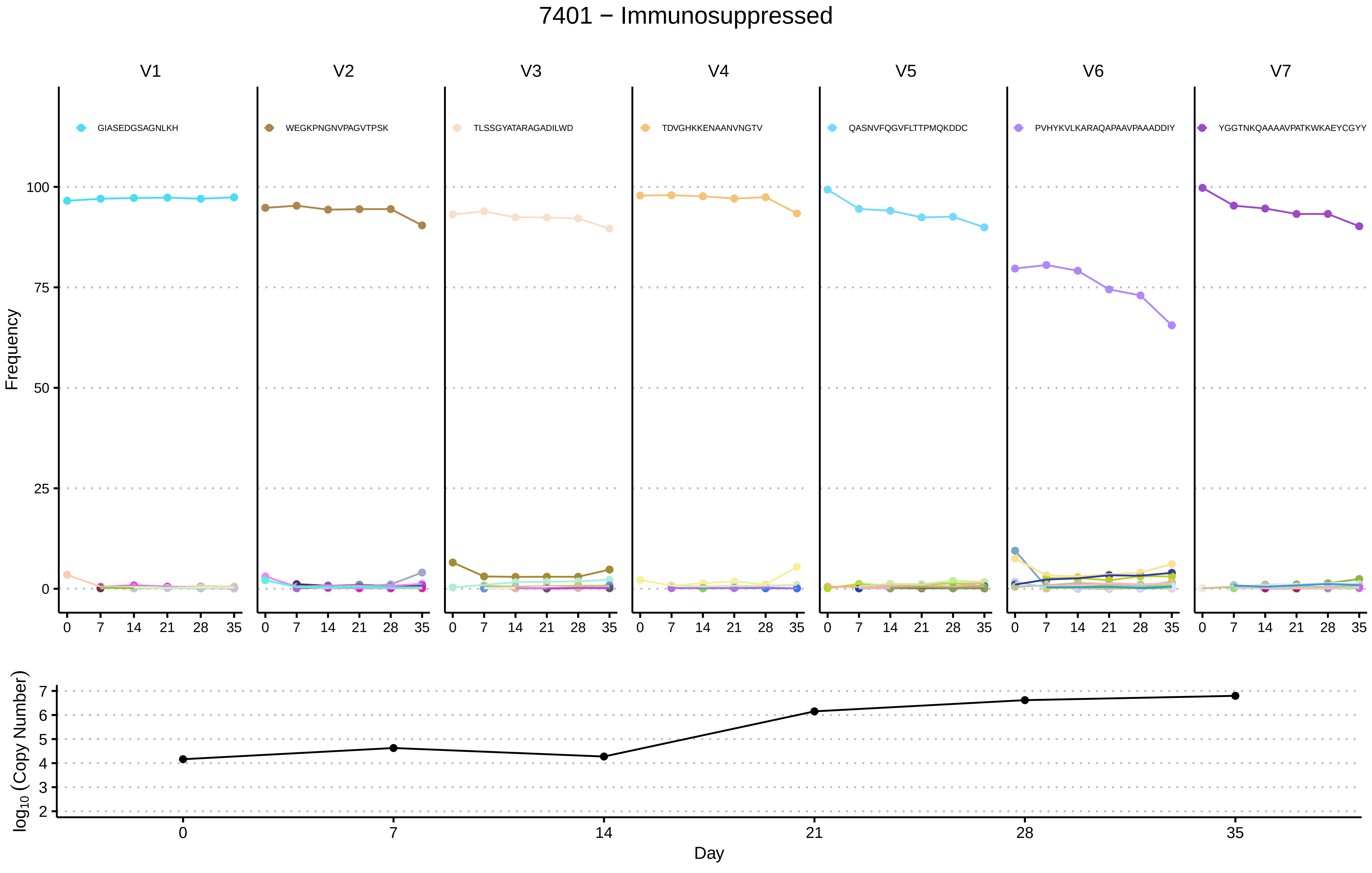

7401 − Immunosuppressed
V1
V2
V3
V4
V5
V6
V7
GIASEDGSAGNLKH
WEGKPNGNVPAGVTPSK
TLSSGYATARAGADILWD
TDVGHKKENAANVNGTV
QASNVFQGVFLTTPMQKDDC
PVHYKVLKARAQAPAAVPAAADDIY
YGGTNKQAAAAVPATKWKAEYCGYY
Frequency
100
75
50
25
0
0 7 14 21 28 35
log10 (Copy Number)
7
6
5
4
3
2
0
7
14
21
28
35
Day

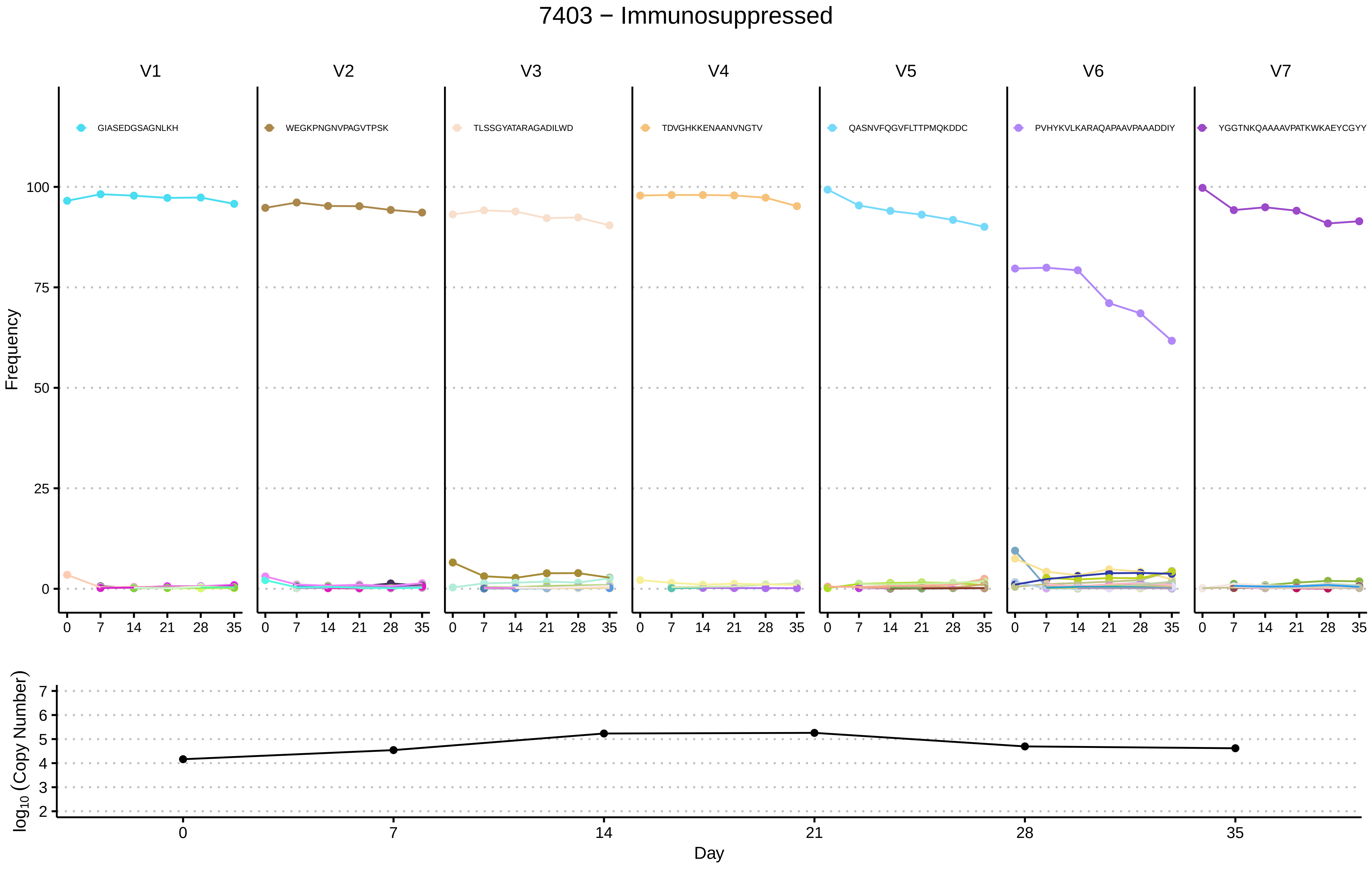

7403 − Immunosuppressed
V1
V2
V3
V4
V5
V6
V7
GIASEDGSAGNLKH
WEGKPNGNVPAGVTPSK
TLSSGYATARAGADILWD
TDVGHKKENAANVNGTV
QASNVFQGVFLTTPMQKDDC
PVHYKVLKARAQAPAAVPAAADDIY
YGGTNKQAAAAVPATKWKAEYCGYY
Frequency
log10 (Copy Number)
Day

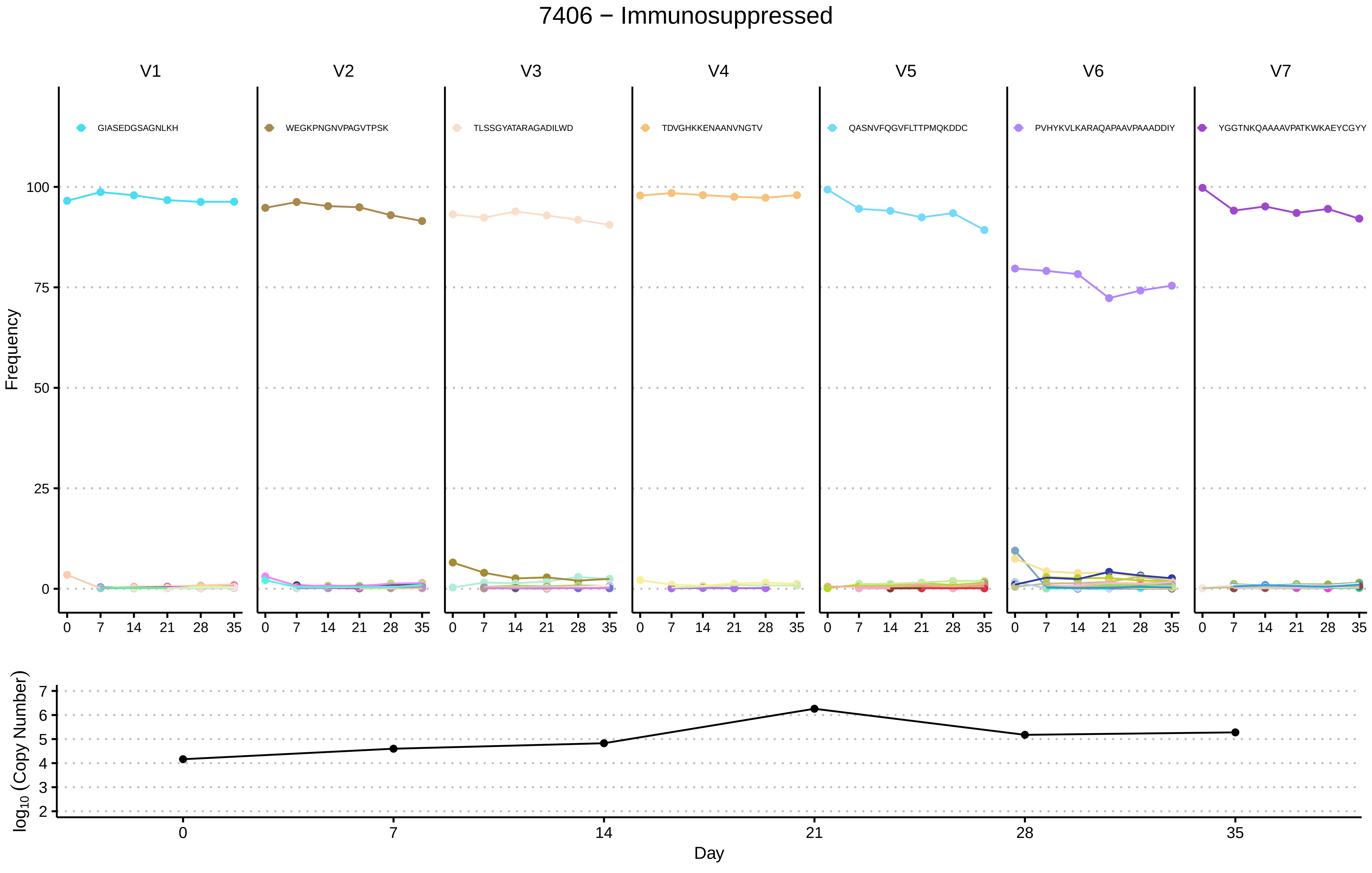

7406 − Immunosuppressed
V1
V2
V3
V4
V5
V6
V7
GIASEDGSAGNLKH
WEGKPNGNVPAGVTPSK
TLSSGYATARAGADILWD
TDVGHKKENAANVNGTV
QASNVFQGVFLTTPMQKDDC
PVHYKVLKARAQAPAAVPAAADDIY
YGGTNKQAAAAVPATKWKAEYCGYY
Frequency
0
25
50
75
100
0 7 14 21 28 35
log10 (Copy Number)
2
3
4
5
6
7
Day
