# Supplementary material for: Longitudinal TprK profiling of in vivo and in vitro-propagated Treponema pallidum subsp. pallidum reveals accumulation of antigenic variants in absence of immune pressure
Source: PLoS Negl Trop Dis. 2021 Sep 7;15(9):e0009753. doi: 10.1371/journal.pntd.0009753 (PMC8480903; doi:10.1371/journal.pntd.0009753)
Supplement: S5 Fig — On the left are sequences constructed with the longest possible donor site segments. Paired on the right are the same sequences following the internal repeat structure rules. Donor sites are represented as labeled color-coded arrows. The first segment of the sequences on the right can be represented by any known V1 donor site. GCAT repeats are highlighted in pink. (PDF) [file pntd.0009753.s008.pdf]

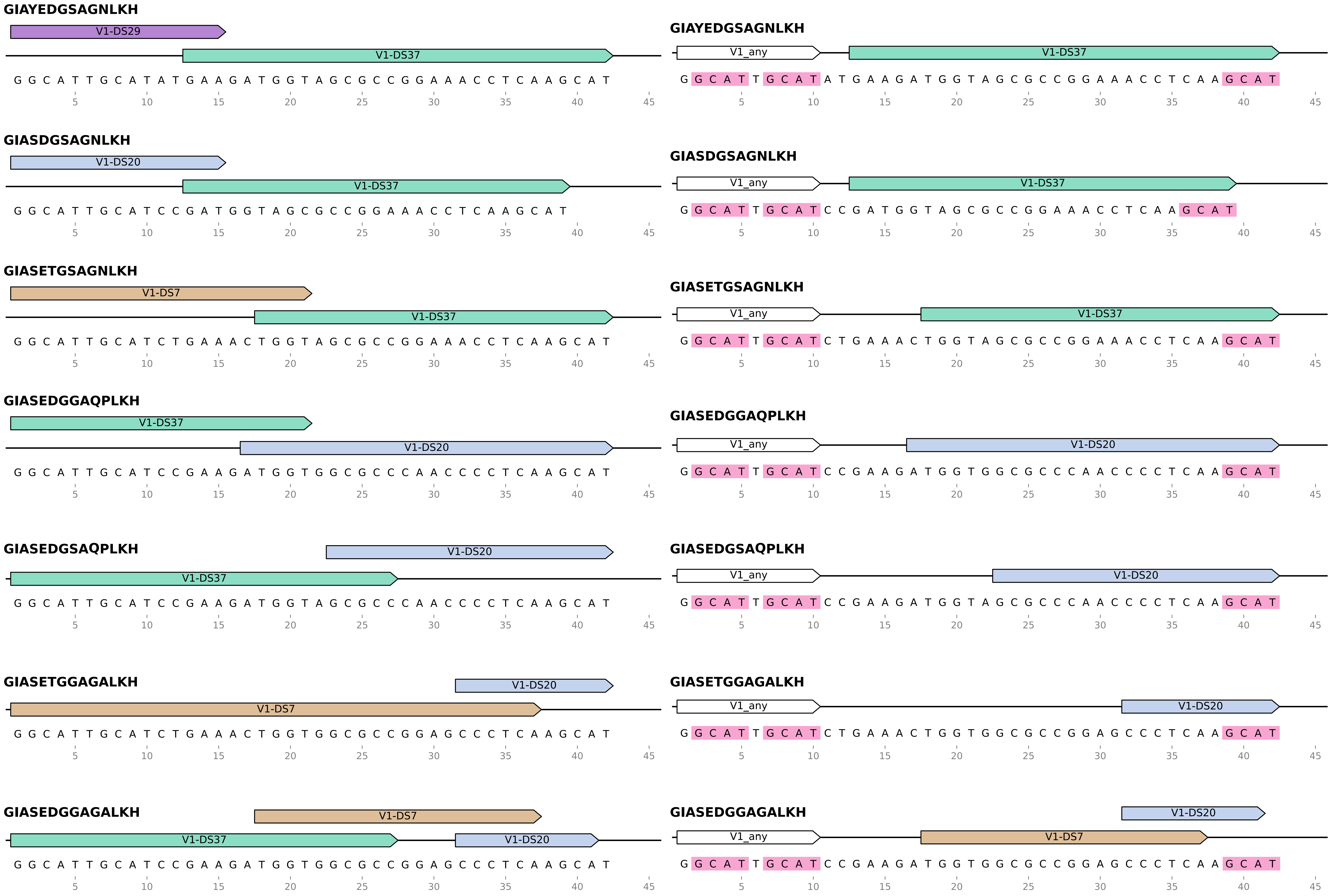
GIAYEDGSAGNLKH
V1-DS29
V1-DS37
G G C A T T G C A T A T G A A G A T G G T A G C G C C G G A A A C C T C A A G C A T
GIASDGSAGNLKH
V1-DS20
V1-DS37
G G C A T T G C A T C C G A T G G T A G C G C C G G A A A C C T C A A G C A T
GIASETGSAGNLKH
V1-DS7
V1-DS37
G G C A T T G C A T C T G A A A C T G G T A G C G C C G G A A A C C T C A A G C A T
GIASEDGGAQPLKH
V1-DS37
V1-DS20
G G C A T T G C A T C C G A A G A T G G T G G C G C C C A A C C C C T C A A G C A T
GIASEDGSAQPLKH
V1-DS20
V1-DS37
G G C A T T G C A T C C G A A G A T G G T A G C G C C C A A C C C C T C A A G C A T
GIASETGGAGALKH
V1-DS20
V1-DS7
G G C A T T G C A T C T G A A A C T G G T G G C G C C G G A G C C C T C A A G C A T
GIASEDGGAGALKH
V1-DS7
V1-DS37
V1-DS20
G G C A T T G C A T C C G A A G A T G G T G G C G C C G G A G C C C T C A A G C A T
GIAYEDGSAGNLKH
V1_any
V1-DS37
G G C A T T G C A T A T G A A G A T G G T A G C G C C G G A A A C C T C A A G C A T
GIASDGSAGNLKH
V1_any
V1-DS37
G G C A T T G C A T C C G A T G G T A G C G C C G G A A A C C T C A A G C A T
GIASETGSAGNLKH
V1_any
V1-DS37
G G C A T T G C A T C T G A A A C T G G T A G C G C C G G A A A C C T C A A G C A T
GIASEDGGAQPLKH
V1_any
V1-DS20
G G C A T T G C A T C C G A A G A T G G T G G C G C C C A A C C C C T C A A G C A T
GIASEDGSAQPLKH
V1_any
V1-DS20
G G C A T T G C A T C C G A A G A T G G T A G C G C C C A A C C C C T C A A G C A T
GIASETGGAGALKH
V1_any
V1-DS20
G G C A T T G C A T C T G A A A C T G G T G G C G C C G G A G C C C T C A A G C A T
GIASEDGGAGALKH
V1-DS20
V1_any
V1-DS7
G G C A T T G C A T C C G A A G A T G G T G G C G C C G G A G C C C T C A A G C A T
